# A Tailored Approach for Appendicular Impending and Pathologic Fractures in Solid Cancer Metastases

**DOI:** 10.3390/cancers14040893

**Published:** 2022-02-11

**Authors:** Joaquim Soares do Brito, Raquel Lopes-Brás, André Abrunhosa-Branquinho, Isabel Fernandes, Inês Gomes, Sandra Casimiro, Luís Costa

**Affiliations:** 1 Department of Orthopedics, Centro Hospitalar Universitário Lisboa Norte, 1649-028 Lisbon, Portugal; 2 Department of Medical Oncology, Centro Hospitalar Universitário Lisboa Norte, 1649-028 Lisbon, Portugal; raquel.bras@chln.min-saude.pt (R.L.-B.); 15673@chln.min-saude.pt (I.F.); 3 Department of Radiotherapy, Centro Hospitalar Universitário Lisboa Norte, 1649-028 Lisbon, Portugal; andre.branquinho@chln.min-saude.pt; 4 Luis Costa Laboratory, Instituto de Medicina Molecular—João Lobo Antunes, Faculdade de Medicina de Lisboa, 1649-028 Lisbon, Portugal; ines.gomes@medicina.ulisboa.pt (I.G.); scasimiro@medicina.ulisboa.pt (S.C.)

**Keywords:** bone metastases, clinical management, impending fracture, pathologic fracture, tailored approach

## Abstract

**Simple Summary:**

Patients with bone metastases often suffer with complications, such as bone fractures, which have a substantial negative impact on clinical outcomes. To optimize clinical results, a tailored approach should be defined for managing impending or pathologic fractures in each individual case. The ability to control systemic disease, the extent, location and nature of bone metastases, and the biology of the underlying tumor, are the main factors that will define the strategy to follow.

**Abstract:**

Advances in medical and surgical treatment have played a major role in increasing the survival rates of cancer patients with metastatic bone disease. The clinical course of patients with bone metastases is often impaired by bone complications, such as bone fractures, which have a substantial negative impact on clinical outcomes. To optimize clinical results and prevent a detrimental impact on patients’ health, a tailored approach should be defined for any given patient. The optimal management of impending or pathologic fractures is unknown and relies on a multidisciplinary approach to tailor clinical decisions to each individual patient. The ability to control systemic disease, the extent, location and nature of bone metastases, and the biology of the underlying tumor, are the main factors that will define the strategy to follow. The present review covers the most recent data regarding impending and pathologic fractures in patients with bone metastases, and discusses the medical and surgical management of patients presenting with metastatic bone disease in different clinical settings.

## 1. Introduction

The skeleton is a common site of metastatic disease in patients with advanced solid tumors, particularly breast, prostate, renal, thyroid and lung tumors [1]. Medical advances over the last decades have been responsible for a progressive and remarkable increase in the survival of patients with cancer, and thus in the number of patients living with bone metastases (BM) [1]. The greater number of patients with BM also increases the overall number of skeletal-related events (SREs), with a relevant detrimental impact on clinical outcomes [2,3]. Among SREs, pathologic fractures are particularly pernicious, increasing morbidity, reducing quality of life, and shortening overall survival in these patients [2,3,4,5,6]. Additionally, the economic and social burden associated with pathologic fractures is high [7,8]. As such, the ideal approach is to prevent fractures by treating the impending fracture. However, its diagnosis is often challenging, and scores used in clinical practice frequently overestimate the risk of fracture, leading to unnecessary interventions [9,10]. In addition, the management of pathologic or impending fractures can be complex, with earlier orthopedic evaluation potentially precluding the need for complex procedures [11].

Depending on the tumor of origin, the approach to BM may differ, not only regarding systemic therapy, but also in indications for surgery, radiotherapy (RT) and bone-targeted therapies.

This review focuses on the need for personalized approaches for appendicular impending and pathologic fractures associated with BM, according to histologic subtype, based on the available clinical evidence.

## 2. Epidemiology of Metastatic Bone Disease

More than 75% of patients with metastatic cancer have bone involvement at the time of death, with the spine, ribs, pelvis and long bones being the most common sites of BM [12,13]. Few epidemiologic studies on skeletal metastases are available, but according to data from the United States (US), approximately 280,000 adults live with metastatic bone disease, with breast and prostate cancer being the dominant tumors [14,15]. This lack of data is even more striking regarding pathologic fractures. Nonetheless, among patients with metastatic carcinomas, US estimates indicate a 20% rate of relevant clinical symptoms and a 10% rate of pathologic fractures [15]. Therefore, thousands of pathologic fractures per year would be expected in the US population alone [16]. These numbers could be even higher, as studies suggest that 1.4 million people are diagnosed with cancer every year in the country, and half of the patients have cancers that frequently metastasize to the bone [16]. In 2018, Nikkel et al. reported 17,313 hospitalizations due to pathologic fractures and 12,770 due to non-pathologic fractures (related to metastases, secondary to minor trauma) in patients with metastatic bone disease between 2003 and 2010 in the US. In this report, kidney cancer had the highest hospitalization rate for pathologic fractures [17]. In 2011, Jensen et al. reported a 45% rate of SREs, including pathologic fractures, among patients with BM in Denmark [18]. Other studies have reported data on the incidence of pathologic fractures among patients with BM and SREs, with pathologic fracture rates of 16% in breast carcinoma [19], 22.1% in prostate cancer [20], 21.6% in lung cancer [21], and 42% in renal cancer [22]. Given the high incidence and prevalence reported for some of these tumors, the real incidence of pathologic fractures will predictably become even more relevant during the 21st century. 

An increasingly relevant discussion is emerging regarding BM and the prognostic impact of pathologic fractures on survival. Oefelein et al. conducted a study on 195 prostate cancer patients and reported a negative correlation between bone fractures and overall survival [23]. Another study by Saad et al., including 3059 patients with multiple myeloma and various solid tumors, showed that pathologic fractures were related to an increased risk of death [24]. Additionally, Yong et al. conducted a population-based cohort study and reported that, among 2216 patients with BM, the median survival was far superior in patients without SREs compared with those with SREs [2]. However, although several studies have reported increased mortality rates associated with SREs or pathologic fractures in solid tumors, the causes for this correlation are not entirely understood. Some of the factors thought to be involved include mortality due to surgery for pathologic fractures, and increased risk of thromboembolic events (namely, deep vein thrombosis and secondary pulmonary embolism), to which diminished mobility also contributes [24]. Altogether, these data highlight the importance of preventing pathologic fractures.

## 3. Physiopathology of Osteolysis and Bone Fragility 

Although low bone mineral density (BMD) is a strong risk factor for fractures, bone size, shape and microarchitecture may also influence skeletal fragility [25]. Therefore, the concept of bone quality has evolved to include toughness, strength, resistance to failure, load-bearing ability, geometry, micro-damage and bone mass [25]. Altogether, these characteristics are summarized as bone architecture, turnover, and mineralization, which depend on a tightly controlled skeletal homeostasis. Unbalanced bone turnover elicits bone fragility and weakens the skeleton, increasing fracture risk whenever the force applied to the bone exceeds its load-bearing capacity. 

**Figure 1:**
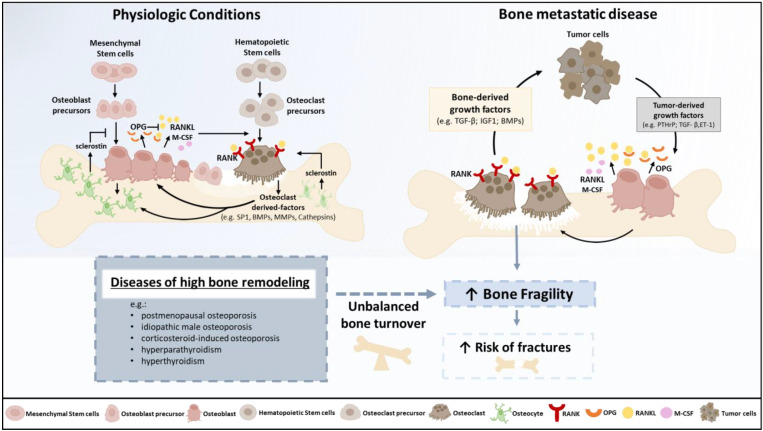
Bone remodeling in physiologic conditions and in metastatic disease. In physiologic conditions, bone resorption and bone formation are perfectly coordinated by bone cells (osteoclasts, osteoblasts and osteocytes), regulated by hormones, like parathyroid hormone (PTH) and 1,25-dihydroxyvitamin-D3, paracrine growth factors, and cytokines [26,33], to guarantee skeletal homeostasis. Bone-resorbing osteoclasts originate from hematopoietic stem cells, and bone-forming osteoblasts differentiate from mesenchymal precursors. The main bone remodeling regulator is the receptor activator of the nuclear factor-κB (RANK)/RANK ligand (RANKL)/osteoprotegerin (OPG) axis. Osteoblasts release osteoclastogenic factors, such as the macrophage-colony-stimulating factor (M-CSF) and RANKL. Osteocytes also contribute to the bone remodeling balance by inhibiting osteoblast or promoting osteoclast differentiation. In metastatic disease, bone homeostasis is disrupted, and bone remodeling is unbalanced towards osteolysis. Tumor cells secrete growth factors that stimulate osteoclast differentiation and activity, like parathyroid hormone-related peptide (PTHrP) and interleukin-11 (IL-11). The bone destruction process releases bone-derived growth factors supporting tumor cell growth, like transforming growth factor β (TGFβ) and bone morphogenetic proteins (BMPs), creating a vicious cycle. This unbalanced bone turnover elicits or increases bone fragility, increasing fracture risk.

The coupling between bone resorption and formation is a multifaceted process, as reviewed by Sims and Martin [26]. The key physiological controller of osteoclast formation and activity is the RANKL/RANK/osteoprotegerin (OPG) axis, the RANKL decoy receptor [27]. The pivotal roles of the RANKL/RANK/OPG system in osteoclast differentiation and function, and of dual signaling in bone resorption and formation coupling, were recently reviewed by Yasuda [28]. Pathologic fractures in the context of BM are the result of de novo or exacerbated bone fragility due to unbalanced bone remodeling towards osteolysis (Figure 1). Bone osteolysis releases multiple growth factors that fuel tumor growth, rendering the so-called ‘vicious cycle’ of BM, in which the expansion of cancer metastases within bone involves paracrine signaling between tumor cells and normal cells in the bone microenvironment [29]. Several diseases of high bone remodeling—like postmenopausal osteoporosis, idiopathic male osteoporosis, corticosteroid-induced osteoporosis, hyperparathyroidism and hyperthyroidism, all linked to bone fragility [30]—may increase the risk of pathologic fractures in cancer patients. BM may have different radiologic presentations, spanning from pure osteolytic, like in renal cell carcinoma and multiple myeloma, to osteoblastic, like in prostate cancer. However, in most cases, the phenotype is mixed, and bone fragility is increased [31,32]. 

The bone is a favorable metastatic niche for different cancers, including breast (70%), prostate (85%), lung (40%) and kidney (40%) cancers and multiple myeloma (95%) [34]. The axial skeleton is most affected, reflecting the role of the red bone marrow as an active microenvironment that promotes cellular growth [31]. Circulating cancer cells enter the bone through the wide-channeled sinusoids of the bone marrow cavity, invade the marrow stroma, and travel to the endosteal bone surface, in a process mediated by stromal cell-derived factor 1 (SDF1), also known as the C-X-C motif chemokine 12 (CXCL12)—C-X-C chemokine receptor type 4 (CXCR4) pathway [35,36]. Activated RANK–RANKL signaling has also been implicated in bone colonization during metastatic development in prostate and breast cancers [37,38,39]. Following bone seeding, disseminated tumor cells may undergo dormancy, which can last decades, with signals triggering tumor growth still unknown. Conversely, the subsequent bone colonization step, which accounts for the critical transition between micro and macrometastases, is well understood [40,41]. Physical factors within the bone microenvironment, namely hypoxia, acid pH and extracellular Ca^2+^, and bone-derived growth factors, such as transforming growth factor β (TGFβ) and insulin growth factor (IGF), activate the tumor expression of osteoblast-stimulatory factors, including bone morphogenetic proteins (BMPs), VEGF and endothelin 1 (ET-1). Osteoclast-stimulatory factors, like parathyroid hormone-related peptide (PTHrP) and interleukin 11 (IL-11), are also increased and up-regulate RANKL on early osteoblasts, stimulating bone resorption via the RANK receptor on osteoclasts. The exacerbated release of matrix proteases by osteoclasts, like matrix metalloproteases (MMPs) and cathepsins, further increases the release of matrix-trapped factors and tumor growth [40,41].

Factors like ET-1 are particularly relevant for the development of osteoblastic metastases, stimulating osteoblast proliferation and differentiation via the endothelin A receptor (ETR) and activating Wnt signaling [42,43], whereas osteolytic metastases are mainly related to PTHrP and IL-11-mediated RANKL upregulation [44] and Jagged1-induced fusion of osteoclast precursors [45].

## 4. Impending and Pathologic Fractures Associated with Solid Metastases

The accurate risk assessment of pathologic fractures (impending fractures) is challenging and still lacks an optimal method. Fidler et al. estimated a 2.3% pathologic fracture risk in patients with less than 50% of cortical involvement [46]. On the other hand, a 75% cortical involvement correlated with 80% of pathologic fractures [46]. In another study, Menck et al. estimated that more than 90% of patients with at least a 0.6 ratio between metastasis width and bone diameter eventually suffered fractures [47]. Subsequently, Harrington et al. proposed criteria to guide clinical decision making, including cortical involvement of 50%, metastasis length superior to 2.5 mm, and persistent pain even after RT, introducing for the first time a symptomatic factor (pain) into the equation, to estimate the pathologic fracture risk [48]. In the late 1980’s, Mirels developed a 12-point score to estimate the pathologic fracture risk (Table 1) based on the following four items: bone involved, anatomic region, radiologic presentation of metastases (lytic, sclerotic or mixed), and presence of pain under physiologic load [9]. The pathologic fracture risk was estimated at 33% for a score ≥9, suggesting the need for preventative surgical stabilization. However, Damron et al. exposed the low specificity of this score, which in practice represented an overestimation of the need for surgical stabilization [10].

More recently, van der Wal et al. conducted a study to evaluate the feasibility of using bone axial cortical involvement by metastases using a 30 mm threshold on conventional radiographs to assess the fracture risk in femoral BM [49]. The authors observed that patients with metastases with more than 30 mm of axial cortical involvement had a 5.3-times higher fracture risk compared with patients with smaller lesions. Based on these findings, they considered the 30 mm axial cortical involvement criteria in femoral metastases as an adequate tool for assessing pathologic fracture risk [49]. Additionally, new evaluations based on CT imaging using the finite element method, enabled a more accurate estimation of fracture risk in the presence of metastases [50,51]. Nonetheless, this technology is far from being easily accessible in clinical practice. Therefore, more practical and replicable systems are desirable to estimate the real and accurate risk of pathologic fractures in patients with metastatic bone disease. Currently, tools such as the Mirels’ score, together with accurate patient clinical evaluation and clinical sense, are the mainstay for accurate decisions on how to proceed. 

## 5. The Treatment of Impending and Pathologic Fractures Secondary to Bone Metastases

The treatment of BM associated with impending or actual pathologic fractures currently comprises of surgery, external beam radiotherapy (EBRT), bone-targeted agents (BTAs), or different associations of the three modalities. Still, treatment is greatly dependent on tumor histotype and staging, and the main goal in the management of patients with metastatic bone disease is ultimately pain relief and the improvement of quality of life [31]. 

EBRT is usually an option for the prevention of bone fractures and/or spinal cord compression, but most of the supporting evidence focuses on the treatment of appendicular or spinal bone metastases [52,53]. Most EBRT clinical trial endpoints focus on pain relief or, in cases of spinal cord compression, mobility recovery. EBRT is not indicated as monotherapy where there is a high risk of fractures or mechanical instability, and if combinations with other treatments (e.g., surgery) are available. All EBRT prescriptions with conformal 3D radiotherapy technique (3D-CRT) follow the practical rule of short hypofractionation schemes (e.g., single fraction of 8 Gy, 20 Gy in 5 fractions, or 30 Gy in 10 fractions). This rule considers patients’ low performance status and short life expectancy, to reduce Radiotherapy Department visits [54,55]. According to meta-analyses, 3D-CRT is associated with a low incidence of pathologic fractures (≤5%, pooled odds ratio [OR] 1.21, 95% confidence interval [CI] 0.76–1.95) and spinal cord compression (≤3%, pooled OR 1.40, 95% CI 0.73–2.67) [56,57,58]. In addition, a trend for these events to occur more often in single- compared to multi-fractionated schemes, was also observed [56,57,58], making the latter preferred for patients at risk of fractures or spinal cord compression. Evidence regarding postoperative 3D-CRT is more robust for spinal metastases. In the study by Patchell et al., 101 patients with spinal cord compression were assessed to see whether decompressive surgical resection before EBRT (30 Gy in 10 fractions) could increase their ability to walk compared to EBRT alone [59]. The interim analysis showed an improvement in this endpoint (84% vs. 57%, OR 6.2, 95% CI 2.0–19.8). Although postoperative 3D-CRT is frequently employed in cases of appendicular metastases, evidence for its use is currently lacking, according to the systematic review by Willeumier et al. [60]. The NCCN task force recommends radiation therapy for 2–3 weeks after surgery, for wound healing and removal of metallic skin staples [61]. Metallic prosthesis is not a contraindication for EBRT, but interferes with procedure planning. Here also, the trend is for the use of multi-fractionated regimens whenever possible.

As stated above, surgery for pathologic and impending fractures is affected by several factors, such as patient prognosis, histology, number of metastases and anatomic location [62]. Despite these considerations, common goals exist, and when a surgical procedure is planned to stabilize a pathologic or impending fracture, it is crucial to obtain a construct with enough stability to allow immediate full weight-bearing, restore function, minimize pain, prevent (if possible) tumor progression, and last beyond expected patient lifetime [63,64]. The surgical options for addressing BM in any given anatomic segment are wide. For long bone metastases, orthopedic surgeons tend to use nails for diaphyseal lesions, plates and screws when the metaphyseal region needs to be addressed, or megaprosthesis in cases in which reconstruction around the articulation cannot be successfully achieved (usually meta-epiphyseal lesions with poor bone stock) [65,66]. Additionally, polymethylmethacrylate (PMMA) is often used as an adjuvant to all implants to improve stability of the final construct [67].

The pelvis has a very particular and challenging anatomy, which is relevant in the context of BM surgery. Some metastases are located at pelvic sites that do not impact pelvic stability and function, while others can impair hip function and weight-bearing ability, like those located in the posterior ilium and acetabulum [68]. As such, the surgical treatment of pelvic metastases is usually reserved for lesions with a high risk of fracture and, in rare cases, for solitary pelvic metastases [68]. Depending on the type, extension, and location, reconstruction is possible using plates, pins, screws and PMMA [69,70]. However, arthroplasty will be required for some cases, and in others, surgery should be avoided due to the low probability of success. In any case, the decision should be patient-tailored.

Despite being the most common bone site for solid tumor metastases, the spine is an anatomic location where BM are also difficult to approach [68]. As previously mentioned regarding pelvic metastases, surgical procedures for spinal metastases can range from relatively simple to extremely complex or even life threatening [71]. Importantly, newer instrumentation systems are now available, which facilitate spine stabilization and reconstruction [72]. Most procedures for spine metastatic disease are performed using these systems, which aim to improve patients’ quality of life and, indirectly, life expectancy. Although there is also a place for complete tumor lesion excision, surgery is more aggressive and has a potentially high rate of serious complications [59,73,74]. Overall, considering the general principles of metastatic bone disease treatment, the surgical decision and techniques employed should always involve a multidisciplinary assessment of the best option in every given clinical situation.

Surgery and RT are by far the preferred treatments for impending and pathologic fractures in the appendicular skeleton. However, some BTAs, such as bisphosphonates and denosumab, can also be considered to prevent new fractures and eventually strengthen the bone after fractures occur (secondary prevention) [75]. Several experimental models of BM have shown that RANKL antagonists prevent tumor-associated osteolysis and significantly reduce skeletal tumor burden [76]. Bisphosphonates inhibit osteoclastic activity by strongly binding to hydroxyapatite on the bone surface and inducing osteoclast apoptosis upon internalization [77]. In clinical trials, bisphosphonates have been shown to diminish the incidence of SREs when used in addition to systemic anticancer therapies, compared to anticancer therapies alone [31]. Denosumab acts by binding to and inhibiting RANKL, leading to a decrease in osteoclast differentiation and activity [78]. Clinical trials (including phase III) comparing denosumab to bisphosphonates (namely, zoledronate (ZA)) in patients with solid tumors and multiple myeloma, have shown that denosumab was at least non-inferior to ZA in preventing SREs (time to first SRE) [31,79,80]. Moreover, in a phase III trial in metastatic breast cancer, denosumab was superior to ZA, in addition to standard anticancer therapies, delaying the time to the first on-study SRE and the time to the first and subsequent (multiple) on-study SREs [81]. Similar results were seen in castration-resistant metastatic prostate cancer [82]. The median time to first on-study SRE was 20.7 months with denosumab, compared with 17.1 months with ZA. In a post hoc analysis of a phase III trial in patients with solid tumors and multiple myeloma with bone metastases, denosumab significantly delayed the time to the first on-study SRE and the time to the first and subsequent SREs, compared to ZA [83]. These results support the use of BTAs in the prevention of SREs (including pathologic fractures) and seem to favor the use of denosumab over ZA.

The combination of RANKL inhibition with hormonal therapy or chemotherapy, resulted in significantly greater skeletal tumor growth inhibition than either single agent alone in breast, prostate and lung cancer models [84]. In addition to mediating tumor-induced bone destruction, RANKL also seems to be involved in tumorigenesis and metastases development [85,86]. This was mostly studied in breast cancer, as the role of RANK/RANKL/OPG in normal breast development is important to understand breast cancer tumorigenesis [87]. The activation of progesterone receptor-positive mammary epithelial cells by progesterone leads to RANKL expression, thus resulting in the proliferation of neighboring RANK+ mammary epithelial progenitor cells. Through the induction of R-spondin, RANKL can also elicit the proliferation of RANK-positive ductal epithelial cells. Postmenopausal women with higher levels of RANKL and progesterone are at increased risk of breast cancer [88], and higher levels of serum RANKL seem to be associated with a higher risk of estrogen receptor-positive breast cancer [87]. Additionally, the RANKL/RANK/OPG axis plays a role in the progression of solid tumors, by inducing the loss of apical–basal polarity, promoting tumor cell migration and invasion, and stimulating tumor neovascularization—processes involved in metastasis development [86,89,90]. The addition of either bisphosphonates or denosumab to the treatment regimen can be useful for patients with pathologic fractures. Current international clinical guidelines advocate the use of BTAs (such as ZA or denosumab) concomitantly to sequential systemic treatments in the setting of bone metastases, as the control of the underlying disease is important for preventing the further development of bone metastases and reducing the risk of pathologic fractures and other SREs, such as bone pain [31,75,91,92]. However, issues regarding choice of BTA, the dosing schedule and the duration of treatment are not yet well established [93]. Besides these factors, additional questions rise when discussing the use of bisphosphonates and denosumab, namely, the need of a previous oral evaluation and regular follow-up by a dentist (bringing the matter of poor dental care access in many countries to light) [94]. Another consideration to be accounted for is hypocalcemia, an expected adverse effect (patients should start on calcium and vitamin D supplementation) of both bisphosphonates and denosumab, as well as the association of these agents with atypical fractures. To consider also, is the risk of vertebral fracture returning to pretreatment levels upon denosumab suspension and the risk of renal insufficiency with the use of bisphosphonates.

A recent review by Coleman et al. addressed emerging therapeutic agents that could act as bone resorption antagonists [31]. Among those were firstly the mTOR inhibitor everolimus, based on the fact that the osteoclast differentiation factor RANKL signals through the mTOR/p70 S6 kinase axis, making osteoclast activity dependent on the mTOR pathway [95,96]; secondly, cathepsin K inhibitors, since the main osteoclast-derived protease cathepsin K is responsible for digesting collagen type I in bone; thirdly, tyrosine kinase inhibitors, like the MET and VEGFR inhibitor cabozantinib, as MET and VEGF signaling are important for osteoclast and osteoblast growth and activity [97]; and fourthly, hormone therapy (e.g., abiraterone), among other drug classes. Clinical studies are still ongoing [31]. Regarding the use of immunotherapy, a recent review by Liu et al. explored the role of immunotherapy in common solid malignancies and the possible clinical application of immune checkpoint inhibitors in combination with RANKL inhibitors or the VEGFR inhibitor to counteract the immunosuppressive microenvironment in bone metastases and achieve better clinical outcomes [98]. With new medical approaches with targeted therapies and immunotherapy, survival is expected to improve along with quality of life in advanced disease.

## 6. The Management of Appendicular Impending and Pathologic Fractures Based on Tumor Histology

A multidisciplinary approach to managing BM and impending and pathologic fractures is paramount in order to find the best treatment strategy [99]. Cancer boards focusing on musculoskeletal oncology in general and bone metastases in particular, seems to deliver a higher probability of successful decisions [100,101]. Following these principles, we developed a musculoskeletal oncology board meeting at our institution, which is held twice a month, where patients with bone metastases are discussed and decisions made in a patient-centered fashion.

Among several factors affecting the management of appendicular impending or pathologic fractures, tumor histology is of tremendous relevance (Table 2). The vast majority of BM occur in the setting of breast, prostate, lung, renal and thyroid carcinomas [1]. 

### 6.1. Breast Cancer Metastases

**Figure 2:**
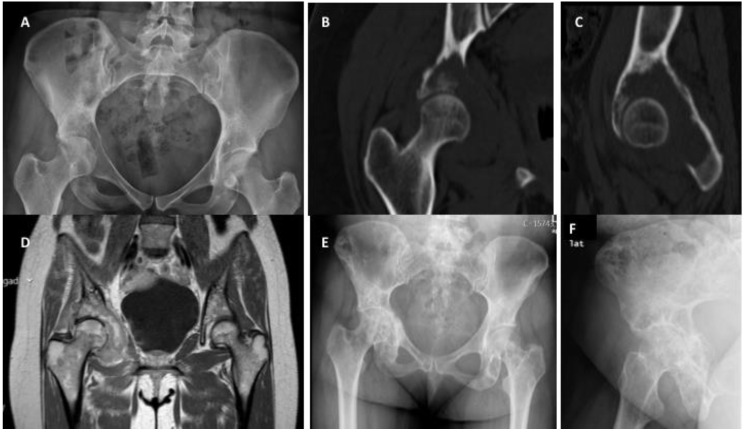
Right acetabular impending fracture in a female patient with breast cancer and multimetastatic bone disease: (**A**) anteroposterior pelvic radiograph showing a lytic lesion located in the right acetabulum, (**B**) pelvic coronal computed tomography showing in more detail the lytic bone metastasis on the right acetabulum, visible in plain radiograph, (**C**) sagittal computed tomography showing a detailed characterization of the lytic bone metastasis on the right acetabulum, (**D**) magnetic resonance coronal image showing the extension of breast cancer metastasis, occupying the right acetabulum, (**E**) pelvic anteroposterior radiograph showing an exceptional result three months after radiation and denosumab treatment of the impending fracture in (**A**), (**F**) alar view of the right acetabulum after radiation and denosumab treatment.

Breast cancer is among the carcinomas most frequently presenting BM. Breast BM typically exhibit lytic or mixed lesions [102]. For patients with solitary or oligometastatic disease (an unusual presentation), the treatment goal should be wide resection of metastases to enable long-term survival without evidence of disease [103,104]. Most often, however, patients with breast cancer BM present with multimetastatic disease. In this setting, non-invasive treatments, such as RT and BTAs without surgery, are plausible options, even with impending fractures (Figure 2) and when surgical treatment is particularly difficult. Nonetheless, this approach is always more straightforward for lesions presenting in the upper limbs compared with the lower limbs. 

**Figure 3:**
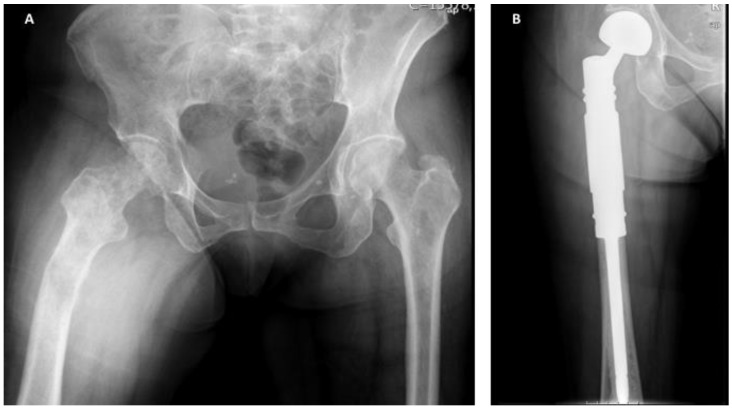
Extensive breast cancer bone metastasis in a female patient with multimetastatic bone disease: (**A**) anteroposterior pelvic radiograph showing a pathologic fracture in the right femur, not amenable for reconstruction due to extension of the metastatic bone disease, (**B**) immediate postoperative radiograph showing anteroposterior view of the right femur with a bipolar tumoral hip megaprosthesis, used to provide stability and pain relief. After surgery, the patient underwent local radiotherapy and bone-targeted agents.

For pathologic fractures, and if stable bone reconstruction is achievable, osteosynthesis followed by RT and BTAs represents the first option. However, if stable reconstruction is not possible, bone segment replacement followed by EBRT and BTAs should be preferred (Figure 3).

### 6.2. Prostate Cancer Metastases

Prostate cancer often presents with BM, which are typically osteoblastic [105]. The process behind the development of osteoblastic metastases is still largely unknown but it seems to be related to the tumor production of osteoblastic factors, such as ET-1, growth differentiation factor 15, and bone morphogenic proteins [106]. Due to their osteoblastic nature, prostate BM display a lower probability of generating impending or pathologic fractures compared to other tumors, which is reflected in score systems, such as the one presented by Mirels (Table 1) [9]. 

**Figure 4:**
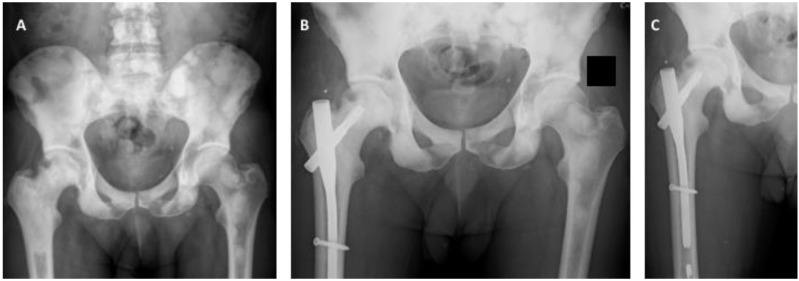
(**A**) Anteroposterior pelvic radiograph showing countless prostate carcinoma metastases around the lower spine, pelvis and proximal femur. Extensive invasion of the pertrochanteric area of the right proximal femur associated with mechanical pain can also be observed. (**B**) Postoperative anteroposterior radiograph of the pelvis after surgical stabilization of the right proximal femur metastasis using a cephalomedullary nail. (**C**) Anteroposterior radiograph of the right hip after surgical stabilization of prostate cancer metastasis in the proximal femur. A broken drill is visible in the medullary canal.

Resection is the strategy of choice for patients presenting with solitary or oligometastatic disease, but similarly to breast carcinoma, these cases are extremely rare. For the more common cases, where patients have multimetastatic disease, the osteoblastic nature of metastases allows a watchful waiting approach, which is even more suitable for non-weight-bearing segments. Nonetheless, for impending (Mirels ≥ 9) or pathologic fractures, surgical stabilization or bone segment replacement should be performed as soon as possible. However, these procedures are extremely difficult due to the hardness and rigidity of prostate metastases, which can originate drill or screw fractures during the surgical process (Figure 4). 

### 6.3. Lung Cancer Metastases

**Figure 5:**
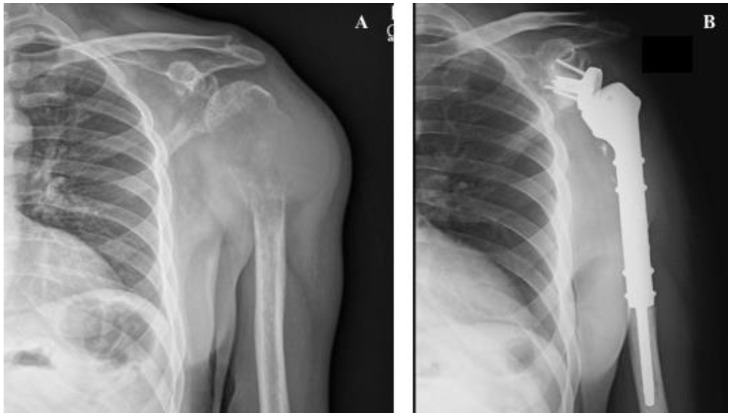
Shoulder megaprosthesis in a solitary proximal humerus lung metastasis: (**A**) preoperative image showing the left humerus with pathologic fracture secondary to lung carcinoma. An important soft tissue component is visible. (**B**) Postoperative image showing shoulder reverse megaprosthesis.

Lung carcinomas typically present with extensive metastatic disease and extremely osteolytic and destructive BM [107]. Similar to other tumor types, a wide resection should be attempted in the rare cases of patients with solitary metastatic disease. Despite the expected good response to radiation, bone metastases in the setting of lung carcinoma often present with pathologic fractures due to the highly destructive nature of the lesions (Figure 5). This destructive pattern makes reconstructive techniques difficult to execute. Although the same principle is true for impending fractures, radiation combined with BTAs can be attempted in cases with a Mirels score ≤7, and particularly for non-weight-bearing segments, since the probability of progress towards fracture is lower. Nevertheless, RT and additional BTAs should be added after the surgical procedure [108]. 

### 6.4. Renal Cancer Metastases

**Figure 6:**
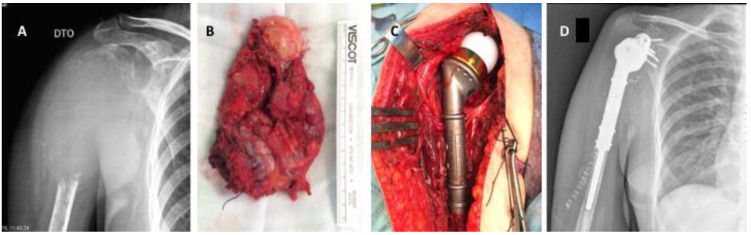
Shoulder megaprosthesis in a solitary kidney cancer metastasis in the proximal humerus: (**A**) preoperative image showing the right humerus with an extensive osteolytic lesion and pathologic fracture secondary to kidney carcinoma. An important soft tissue component is visible. (**B**) Tumor specimen after en bloc resection, (**C**) intraoperative image showing the tumoral megaprosthesis in place, (**D**) immediate postoperative anteroposterior radiograph.

Renal cancer metastases are characterized by extensive lytic, highly vascularized bone lesions with the particular feature of being radiation-resistant [109,110]. Due to these features, renal cancer metastases almost always require surgery, either to provide stabilization or perform substitution of the bone segment or articulation (Figure 6). If open surgery is planned, preoperative embolization is of paramount importance to technically facilitate the procedure and minimize potentially life-threatening blood loss [111].

As with all other carcinomas, patients with solitary or oligometastatic bone disease should undergo total tumor resection, while those with multimetastatic disease are candidates for a less aggressive surgical approach.

### 6.5. Thyroid Cancer Metastases

Similar to renal cancer metastases, thyroid BM are extensive lytic bone lesions, highly vascularized and radiation resistant [112]. Due to these features, surgery is the preferential approach for this histotype, with EBRT and BTAs providing more limited responses.

Due to similar features with renal carcinoma, also for thyroid carcinomas, preoperative embolization is required if open surgery is planned [111], and the same principles regarding localized or multimetastatic disease apply.

## 7. Primary Malignant Bone Tumors and Bone Metastases

Primary malignant bone tumors (PMBT) are extremely rare neoplasms across all ages with an overall estimated incidence between 0.8–0.9 cases per 100,000 per year [113]. While osteosarcoma (OS) and Ewing sarcoma (ES) have a relatively high incidence in young patients (mostly around the second decade of life), chondrosarcoma (CS) will often arise in older ages [113].

Among all PMBT, osteosarcoma is the most common, presenting a higher incidence in adolescents and arising most often in the appendicular skeleton, such as the distal femur, proximal tibia and proximal humerus [114,115]. In a similar fashion, ES will affect mostly children and adolescents, with more than 50% of cases arising in the extremity bones. However, in comparison with OS, ES will present a higher incidence in the pelvis, ribs and vertebrae [115]. CS is the most common malignant bone tumor in adults and arises most frequently in the pelvis and distal and proximal femur, as de novo or as result of the differentiation from pre-existing lesions, such as osteochondroma or enchondroma [113,116]. 

The clinical presentation for PMBT is characterized by bone pain, predominantly at night, swelling and functional impairment, however, these clinical features can be overlapped by any BM or even other diseases, such as osteomyelitis [113]. Nonetheless, BM or bone lesions arising in a multiple myeloma setting are much more common. Additionally, patient’s age will be extremely useful in guiding the diagnosis, since BM and myeloma will be dominant in adulthood after 40 years of age [113]. Conventional radiography (the first imaging exam that should be required in a primary evaluation for bone lesions) will typically show permeative osteolytic bone lesions in BM (with the exception of prostate metastases), while PMBT as OS will often demonstrate a lytic lesion with extensive bone destruction, associated with some sclerotic areas arising from the mineralized osteoid matrix typically produced by the tumor [117,118]. Another aspect to evaluate on conventional radiographs in OS, should be the periosteal reaction, which can be exuberant and with classic radiographic findings, such as the Codman triangle or the sunburst appearance [119]. ES is another example of a bone tumor where an exuberant periostic reaction occurs, often presenting with the appearance of onion layers [119]. CS on the other hand, similarly to OS, will present sclerotic areas on radiographs due to the mineralized cartilage matrix produced by these tumors [119]. BM and PMBT can both present with an associated surrounding soft tissue mass, which will be more easily visible on the magnetic resonance imaging [117]. However, these masses are typically hard in malignant bone tumors due to bone and cartilage matrix production, while they are soft in a BM setting. 

The setup around BM and PMBT regarding clinical and imaging presentation is usually enough to differentiate one from the other. Nonetheless, for solitary BM, a biopsy should always be obtained to establish the proper diagnosis [113,120].

## 8. Discussion

**Figure 7:**
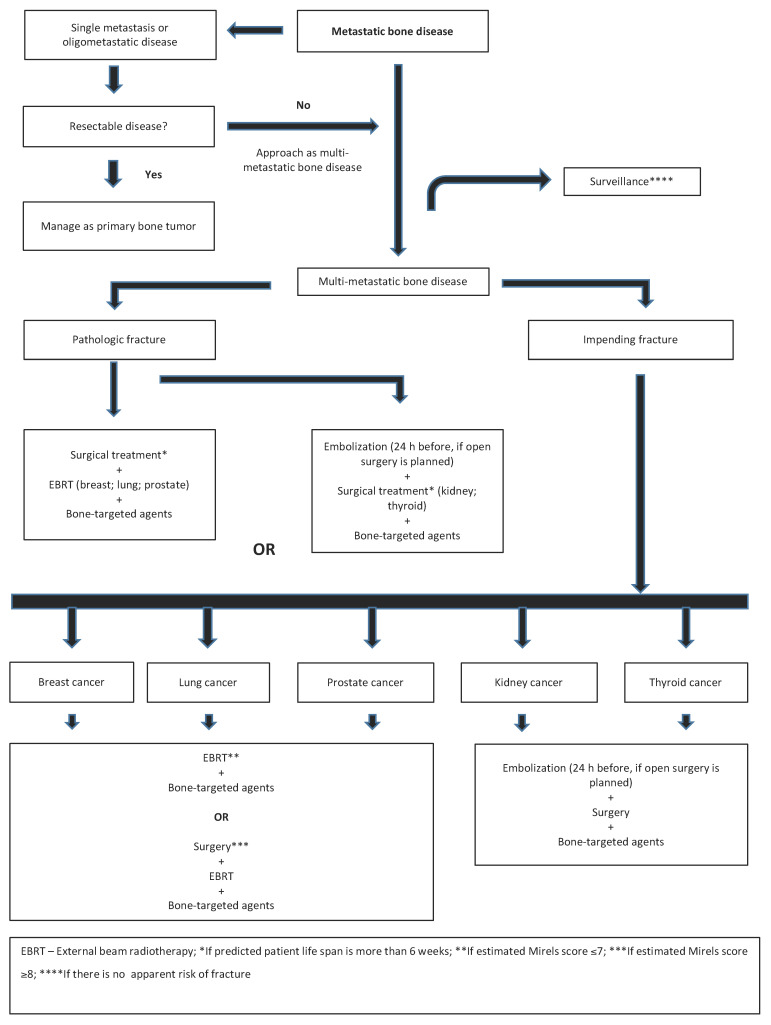
Decision flowchart for patients with appendicular metastatic bone disease.

The skeleton is one of the most frequent sites of metastatic carcinoma dissemination [1]. Although bone involvement usually means incurable disease, a significant number of patients survive for a long period of time [121,122]. These patients are thus more likely to develop SREs, and pathologic fractures in particular [2,3,4,5,6]. Although empirically easy to perform, risk assessment for pathologic fractures is objectively complex. Different authors have sought to develop clinical tools to predict the risk of pathologic fractures and facilitate the clinical decision, but an optimal method remains elusive [9,46,47,48,49,50,51]. Therefore, management decisions for patients with BM are based on clinical data supported by one of several available systems, with good clinical sense also playing a crucial role (Figure 7). Ultimately, the predisposition of cancer patients to develop pathologic fractures is multifactorial. 

BM are directly related to reduced bone strength and hence increased fracture risk, but other factors (e.g., immobility, malnutrition, radiotherapy and chemotherapy) may also enhance the probability of fractures. To add even more uncertainty, factors such as anatomic location and tumor biology, also influence the risk of pathologic fractures, although the extent and degree of that influence remain largely unknown [29,31,34,35,37,40,41].

Therapeutic options are available to prevent the development of pathologic fractures in the setting of metastatic bone disease. Several experimental models of BM have shown that RANKL antagonists prevent tumor-associated osteolysis and effectively reduce tumor skeletal events. Bisphosphonates are pyrophosphate analogues with a strong affinity for divalent metal ions (such as calcium) and bone [123]. These drugs are incorporated into the bone matrix by binding to hydroxyapatite crystals and directly inhibiting osteoclastic activity. Nitrogen-containing bisphosphonates also inhibit farnesyl pyrophosphatase, which is essential for osteoclast function and structural integrity, and for apoptosis prevention, affecting osteoclast differentiation and maturation [124]. Denosumab acts by binding to and inhibiting RANKL activity [125,126]. In both cases, the benefit to pathologic fracture risk has been shown and is established both in the literature and clinical practice.

EBRT and surgery remain the cornerstones of pathologic or impending fracture treatment and are often jointly used [62,63,64]. The treatment approach for BM depends on several variables, with a wide range of solutions available in different clinical settings, which, combined with radiation and medical treatment, provide options for a wide spectrum of patients suffering from metastatic bone disease [67,68]. Among these variables, primary tumor histology plays an important role and should guide these patients’ management. The present article presented an overview of options for a tailored approach for patients with metastatic bone disease, beyond treatments such as chemotherapy, hormone therapy and radionuclides, and based on primary tumors, which is an uncommon strategy in this setting. Besides pathology, tumor biology and genomics is likely to play a more significant role in the medical approach of bone metastases in the coming years, as new medical treatments with targeted therapies (including immunotherapy) may lead to better survival and quality of life outcomes, highlighting the importance of precision oncology. BM from different tumors present specific features, which not only generate different responses to EBRT and BTAs, but also require adjusted surgical management. Further research is ongoing in different medical fields, hopefully improving treatment options for these patients and reinforcing the clinical ability to intervene in the near future.

Cancer patients in general, and those with metastatic bone disease in particular, require a systematic multidisciplinary approach. Despite the exhausting discussion about the need for a multidisciplinary management of patients with BM, orthopedic surgeons are still marginally involved in their treatment. Because of this, patients are often submitted to other therapies rather than being offered the possibility of surgical treatment, associated with a potentially positive impact on their quality of life and survival [127]. However, objective criteria for orthopedic evaluation are difficult to establish. The authors strongly believe that orthopedic surgeons should be involved in every clinical decision regarding BM management, as this offers a better chance for the right treatment at the right timing. An integrated and tailored approach represents the best option towards an optimal decision for patients presenting with BM from different solid tumors. 

## 9. Conclusions

The management of patients with metastatic bone disease has witnessed substantial improvements over the last two decades. Knowledge of the bone metastatic process and biology has grown exponentially, effective medical treatments and new treatment modalities have emerged, surgical techniques have greatly evolved, and radiotherapy has been optimized. 

The successful prevention and management of bone fractures associated with metastatic bone disease in solid tumors relies on several distinct factors, including the ability to control the systemic disease, the extent and location of bone metastases, the nature of metastases within the bone, and the biology of the underlying tumor. Tailoring the approach to each individual patient based on these premises will enable the best patient management and hopefully the best outcomes.

## Figures and Tables

**Table 1:** Mirels’ scoring system for identifying impending fractures: ≥9 high risk for pathologic fracture, >8 intermediate risk for pathologic fracture, ≤7 low risk for pathologic fracture [9].

Parameter	1	2	3
Site	Upper limb	Lower limb	Peritrochanteric
Size	<1/3	1/3 to 2/3	>2/3
Lesion	Blastic	Mixed	Lytic
Pain	Mild	Moderate	Functional

**Table 2:** Expected therapeutic response to radiotherapy, surgery and bone-targeted agents, according to histologic subtype.

Tumor Histology	Breast	Prostate	Lung	Kidney	Thyroid
External beam radiotherapy	+++	+++	+++	+	+
Surgery	++	+	++	+++	+++
Bone-targeted agents	+++	+++	++	++	++

## References

[1] Benca E., Patsch J.M., Mayr W., Pahr D.H., Windhager R. (2016). The insufficiencies of risk analysis of impending pathological fractures in patients with femoral metastases: A literature review. Bone Rep..

[2] Yong M., Jensen A., Jacobsen J.B., Nørgaard M., Fryzek J.P., Sørensen H.T. (2011). Survival in breast cancer patients with bone metastases and skeletal-related events: A population-based cohort study in Denmark (1999–2007). Breast Cancer Res. Treat..

[3] Nørgaard M., Jensen A., Jacobsen J.B., Cetin K., Fryzek J.P., Sørensen H.T. (2010). Skeletal related events, bone metastasis and survival of prostate cancer: A population based cohort study in Denmark (1999 to 2007). J. Urol..

[4] DePuy V., Anstrom K.J., Castel L.D., Schulman K.A., Weinfurt K.P., Saad F. (2007). Effects of skeletal morbidities on longitudinal patient-reported outcomes and survival in patients with metastatic prostate cancer. Supportive Care Cancer.

[5] Weinfurt K.P., Li Y., Castel L.D., Saad F., Timbie J.W., Glendenning G.A., Schulman K.A. (2005). The significance of skeletal-related events for the health-related quality of life of patients with metastatic prostate cancer. Ann. Oncol..

[6] Body J.-J., Pereira J., Sleeboom H., Maniadakis N., Terpos E., Acklin Y.P., Finek J., Gunther O., Hechmati G., Mossman T. (2016). Health resource utilization associated with skeletal-related events: Results from a retrospective European study. Eur. J. Health Econ..

[7] Barlev A., Song X., Ivanov B., Setty V., Chung K. (2010). Payer costs for inpatient treatment of pathologic fracture, surgery to bone, and spinal cord compression among patients with multiple myeloma or bone metastasis secondary to prostate or breast cancer. JMCP.

[8] Blank A.T., Lerman D.M., Patel N.M., Rapp T.B. (2016). Is Prophylactic Intervention More Cost-effective Than the Treatment of Pathologic Fractures in Metastatic Bone Disease?. Clin. Orthop. Relat. Res..

[9] Mirels H. (1989). Metastatic disease in long bones. A proposed scoring system for diagnosing impending pathologic fractures. Clin. Orthop. Relat. Res..

[10] Damron T.A., Morgan H., Prakash D., Grant W., Aronowitz J., Heiner J. (2003). Critical evaluation of Mirels’ rating system for impending pathologic fractures. Clin. Orthop. Relat. Res..

[11] Ashford R.U., Randall R.L., Randall R.L. (2016). Bone Metastases: Epidemiology and Societal Effect. Metastatic Bone Disease: An Integrated Approach to Patient Care.

[12] Coleman R.E. (1997). Skeletal complications of malignancy. Cancer.

[13] Hess K.R., Varadhachary G.R., Taylor S.H., Wei W., Raber M.N., Lenzi R., Abbruzzese J.L. (2006). Metastatic patterns in adenocarcinoma. Cancer.

[14] Li S., Peng Y., Weinhandl E.D., Blaes A.H., Cetin K., Chia V.M., Stryker S., Pinzone J.J., Acquavella J.F., Arneson T.J. (2012). Estimated number of prevalent cases of metastatic bone disease in the US adult population. Clin. Epidemiol..

[15] Zacharia B., Subramaniam D., Joy J. (2018). Skeletal Metastasis—An Epidemiological Study. Indian J. Surg. Oncol..

[16] Jacofsky D.J., Haidukewych G.J. (2004). Management of pathologic fractures of the proximal femur: State of the art. J. Orthop. Trauma.

[17] Nikkel L. (2017). Hospitalizations for fracture in patients with metastatic disease: Primary source lesions in the United States. J. Community Supportive Oncol..

[18] Jensen A.Ø., Jacobsen J.B., Nørgaard M., Yong M., Fryzek J.P., Sørensen H.T. (2011). Incidence of bone metastases and skeletal-related events in breast cancer patients: A population-based cohort study in Denmark. BMC Cancer.

[19] Coleman R.E., Rubens R.D. (1987). The clinical course of bone metastases from breast cancer. Br. J. Cancer.

[20] Saad F., Gleason D.M., Murray R., Tchekmedyian S., Venner P., Lacombe L., Chin J.L., Vinholes J.J., Goas J.A., Chen B. (2002). A randomized, placebo-controlled trial of zoledronic acid in patients with hormone-refractory metastatic prostate carcinoma. J. Natl. Cancer Inst..

[21] Joshi A.D., Carter J.A., Botteman M.F., Kaura S. (2011). Cost-effectiveness of zoledronic acid in the management of skeletal metastases in patients with lung cancer in France, Germany, Portugal, the Netherlands, and the United kingdom. Clin. Ther..

[22] Lipton A., Colombo-Berra A., Bukowski R.M., Rosen L., Zheng M., Urbanowitz G. (2004). Skeletal complications in patients with bone metastases from renal cell carcinoma and therapeutic benefits of zoledronic acid. Clin. Cancer Res..

[23] Oefelein M.G., Ricchiuti V., Conrad W., Resnick M.I. (2002). Skeletal fractures negatively correlate with overall survival in men with prostate cancer. J. Urol..

[24] Saad F., Lipton A., Cook R., Chen Y.M., Smith M., Coleman R. (2007). Pathologic fractures correlate with reduced survival in patients with malignant bone disease. Cancer.

[25] Leali P.T., Muresu F., Melis A., Ruggiu A., Zachos A., Doria C. (2011). Skeletal fragility definition. Clin. Cases Miner. Bone Metab..

[26] Sims N.A., Martin T.J. (2014). Coupling the activities of bone formation and resorption: A multitude of signals within the basic multicellular unit. Bonekey Rep..

[27] Lacey D.L., Timms E., Tan H.L., Kelley M.J., Dunstan C.R., Burgess T., Elliott R., Colombero A., Elliott G., Scully S. (1998). Osteoprotegerin ligand is a cytokine that regulates osteoclast differentiation and activation. Cell.

[28] Yasuda H. (2021). Discovery of the RANKL/RANK/OPG system. J. Bone Miner. Metab..

[29] Kozlow W., Guise T.A. (2005). Breast cancer metastasis to bone: Mechanisms of osteolysis and implications for therapy. J. Mammary Gland Biol. Neoplasia.

[30] Chavassieux P., Seeman E., Delmas P.D. (2007). Insights into material and structural basis of bone fragility from diseases associated with fractures: How determinants of the biomechanical properties of bone are compromised by disease. Endocr. Rev..

[31] Coleman R.E., Croucher P.I., Padhani A.R., Clezardin P., Chow E., Fallon M., Guise T., Colangeli S., Capanna R., Costa L. (2020). Bone metastases. Nat. Rev. Dis. Primers.

[32] Ali S.M., Demers L.M., Leitzel K., Harvey H.A., Clemens D., Mallinak N., Engle L., Chinchilli V., Costa L., Brady C. (2004). Baseline serum NTx levels are prognostic in metastatic breast cancer patients with bone-only metastasis. Ann. Oncol..

[33] Hofbauer L.C., Rachner T.D., Coleman R.E., Jakob F. (2014). Endocrine aspects of bone metastases. Lancet Diabetes Endocrinol..

[34] Coleman R.E. (2006). Clinical features of metastatic bone disease and risk of skeletal morbidity. Clin. Cancer Res..

[35] Wang J., Loberg R., Taichman R.S. (2006). The pivotal role of CXCL12 (SDF-1)/CXCR4 axis in bone metastasis. Cancer Metastasis Rev..

[36] Taichman R.S., Cooper C., Keller E.T., Pienta K.J., Taichman N.S., McCauley L.K. (2002). Use of the stromal cell-derived factor-1/CXCR4 pathway in prostate cancer metastasis to bone. Cancer Res..

[37] Jones D.H., Nakashima T., Sanchez O.H., Kozieradzki I., Komarova S.V., Sarosi I., Morony S., Rubin E., Sarao R., Hojilla C.V. (2006). Regulation of cancer cell migration and bone metastasis by RANKL. Nature.

[38] Chu G.C., Zhau H.E., Wang R., Rogatko A., Feng X., Zayzafoon M., Liu Y., Farach-Carson M.C., You S., Kim J. (2014). RANK- and c-Met-mediated signal network promotes prostate cancer metastatic colonization. Endocr. Relat. Cancer.

[39] Wu X., Li F., Dang L., Liang C., Lu A., Zhang G. (2020). RANKL/RANK System-Based Mechanism for Breast Cancer Bone Metastasis and Related Therapeutic Strategies. Front. Cell Dev. Biol..

[40] Casimiro S., Guise T.A., Chirgwin J. (2009). The critical role of the bone microenvironment in cancer metastases. Mol. Cell. Endocrinol..

[41] Casimiro S., Ferreira A.R., Mansinho A., Alho I., Costa L. (2016). Molecular Mechanisms of Bone Metastasis: Which Targets Came from the Bench to the Bedside?. Int. J. Mol. Sci..

[42] Yin J.J., Mohammad K.S., Kakonen S.M., Harris S., Wu-Wong J.R., Wessale J.L., Padley R.J., Garrett I.R., Chirgwin J.M., Guise T.A. (2003). A causal role for endothelin-1 in the pathogenesis of osteoblastic bone metastases. Proc. Natl. Acad. Sci. USA.

[43] Nelson J.B., Hedican S.P., George D.J., Reddi A.H., Piantadosi S., Eisenberger M.A., Simons J.W. (1995). Identification of endothelin-1 in the pathophysiology of metastatic adenocarcinoma of the prostate. Nat. Med..

[44] Thomas R.J., Guise T.A., Yin J.J., Elliott J., Horwood N.J., Martin T.J., Gillespie M.T. (1999). Breast cancer cells interact with osteoblasts to support osteoclast formation. Endocrinology.

[45] Sethi N., Dai X., Winter C.G., Kang Y. (2011). Tumor-derived JAGGED1 promotes osteolytic bone metastasis of breast cancer by engaging notch signaling in bone cells. Cancer Cell.

[46] Fidler M. (1981). Incidence of fracture through metastases in long bones. Acta Orthop. Scand..

[47] Menck H., Schulze S., Larsen E. (1988). Metastasis size in pathologic femoral fractures. Acta Orthop. Scand..

[48] Harrington K.D. (1986). Impending pathologic fractures from metastatic malignancy: Evaluation and management. Instr. Course Lect..

[49] Van der Wal C., Eggermont F., Fiocco M., Kroon H.M., Ayu O., Slot A., Snyers A., Rozema T., Verdonschot N.J.J., Dijkstra P.D.S. (2020). Axial cortical involvement of metastatic lesions to identify impending femoral fractures; a clinical validation study. Radiother. Oncol..

[50] Liebl H., Garcia E.G., Holzner F., Noel P.B., Burgkart R., Rummeny E.J., Baum T., Bauer J.S. (2015). In-Vivo Assessment of Femoral Bone Strength Using Finite Element Analysis (FEA) Based on Routine MDCT Imaging: A Preliminary Study on Patients with Vertebral Fractures. PLoS ONE.

[51] Koivumäki J.E., Thevenot J., Pulkkinen P., Kuhn V., Link T.M., Eckstein F., Jämsä T. (2012). Ct-based finite element models can be used to estimate experimentally measured failure loads in the proximal femur. Bone.

[52] De Felice F., Piccioli A., Musio D., Tombolini V. (2017). The role of radiation therapy in bone metastases management. Oncotarget.

[53] Agarwal J., Baum R., Hoefnagel C., Hoskin P., Mount Kim E., Mariani G. (2007). Criteria for Palliation of Bone Metastases—Clinical Applications.

[54] Lutz S., Balboni T., Jones J., Lo S., Petit J., Rich S.E., Wong R., Hahn C. (2017). Palliative radiation therapy for bone metastases: Update of an ASTRO Evidence-Based Guideline. Pract. Radiat. Oncol..

[55] Wu J.S., Wong R.K., Lloyd N.S., Johnston M., Bezjak A., Whelan T. (2004). Radiotherapy fractionation for the palliation of uncomplicated painful bone metastases—An evidence-based practice guideline. BMC Cancer.

[56] Chow E., Harris K., Fan G., Tsao M., Sze W.M. (2007). Palliative radiotherapy trials for bone metastases: A systematic review. J. Clin. Oncol..

[57] Chow E., Zeng L., Salvo N., Dennis K., Tsao M., Lutz S. (2012). Update on the systematic review of palliative radiotherapy trials for bone metastases. Clin. Oncol..

[58] Rich S.E., Chow R., Raman S., Liang Zeng K., Lutz S., Lam H., Silva M.F., Chow E. (2018). Update of the systematic review of palliative radiation therapy fractionation for bone metastases. Radiother. Oncol..

[59] Patchell R.A., Tibbs P.A., Regine W.F., Payne R., Saris S., Kryscio R.J., Mohiuddin M., Young B. (2005). Direct decompressive surgical resection in the treatment of spinal cord compression caused by metastatic cancer: A randomised trial. Lancet.

[60] Willeumier J.J., van der Linden Y.M., Dijkstra P.D. (2016). Lack of clinical evidence for postoperative radiotherapy after surgical fixation of impending or actual pathologic fractures in the long bones in patients with cancer; a systematic review. Radiother. Oncol..

[61] Gralow J.R., Biermann J.S., Farooki A., Fornier M.N., Gagel R.F., Kumar R., Litsas G., McKay R., Podoloff D.A., Srinivas S. (2013). NCCN Task Force Report: Bone Health In Cancer Care. J. Natl. Compr. Cancer Netw..

[62] Capanna R., Piccioli A., Di Martino A., Daolio P.A., Ippolito V., Maccauro G., Piana R., Ruggieri P., Gasbarrini A., Spinelli M.S. (2014). Management of long bone metastases: Recommendations from the Italian Orthopaedic Society bone metastasis study group. Expert Rev. Anticancer Ther..

[63] Bickels J., Dadia S., Lidar Z. (2009). Surgical management of metastatic bone disease. J. Bone Jt. Surg..

[64] Katzer A., Meenen N.M., Grabbe F., Rueger J.M. (2002). Surgery of skeletal metastases. Arch. Orthop. Trauma Surg..

[65] Tanaka T., Imanishi J., Charoenlap C., Choong P.F.M. (2016). Intramedullary nailing has sufficient durability for metastatic femoral fractures. World J. Surg. Oncol..

[66] Dijstra S., Wiggers T., van Geel B.N., Boxma H. (1994). Impending and actual pathological fractures in patients with bone metastases of the long bones. A retrospective study of 233 surgically treated fractures. Eur. J. Surg. Acta Chir..

[67] Cheung F.H. (2014). The practicing orthopedic surgeon’s guide to managing long bone metastases. Orthop. Clin. N. Am..

[68] Denaro V., Di Martino A., Piccioli A. (2019). Management of Bone Metastases: A Multidisciplinary Guide.

[69] Müller D.A., Capanna R. (2015). The Surgical Treatment of Pelvic Bone Metastases. Adv. Orthop..

[70] Wunder J.S., Ferguson P.C., Griffin A.M., Pressman A., Bell R.S. (2003). Acetabular metastases: Planning for reconstruction and review of results. Clin. Orthop. Relat. Res..

[71] Tomita K., Kawahara N., Kobayashi T., Yoshida A., Murakami H., Akamaru T. (2001). Surgical strategy for spinal metastases. Spine.

[72] Ibrahim A., Crockard A., Antonietti P., Boriani S., Bünger C., Gasbarrini A., Grejs A., Harms J., Kawahara N., Mazel C. (2008). Does spinal surgery improve the quality of life for those with extradural (spinal) osseous metastases? An international multicenter prospective observational study of 223 patients. Invited submission from the Joint Section Meeting on Disorders of the Spine and Peripheral Nerves, March 2007. J. Neurosurg. Spine.

[73] Bauer H.C., Wedin R. (1995). Survival after surgery for spinal and extremity metastases. Prognostication in 241 patients. Acta Orthop. Scand..

[74] Kieser D.C., Parker J., Reynolds J. (2020). En Bloc Resection of Isolated Spinal Metastasis: A Systematic Review Update. Clin. Spine Surg..

[75] Coleman R., Hadji P., Body J.J., Santini D., Chow E., Terpos E., Oudard S., Bruland Ø., Flamen P., Kurth A. (2020). Bone health in cancer: ESMO Clinical Practice Guidelines. Ann. Oncol..

[76] Clezardin P., Coleman R., Puppo M., Ottewell P., Bonnelye E., Paycha F., Confavreux C.B., Holen I. (2020). Bone Metastasis: Mechanisms, Therapies and Biomarkers. Physiol. Rev..

[77] Singh T., Kaur V., Kumar M., Kaur P., Murthy R.S., Rawal R.K. (2015). The critical role of bisphosphonates to target bone cancer metastasis: An overview. J. Drug Target..

[78] Baron R., Ferrari S., Russell R.G. (2011). Denosumab and bisphosphonates: Different mechanisms of action and effects. Bone.

[79] Raje N., Terpos E., Willenbacher W., Shimizu K., García-Sanz R., Durie B., Legieć W., Krejčí M., Laribi K., Zhu L. (2018). Denosumab versus zoledronic acid in bone disease treatment of newly diagnosed multiple myeloma: An international, double-blind, double-dummy, randomised, controlled, phase 3 study. Lancet Oncol..

[80] Henry D.H., Costa L., Goldwasser F., Hirsh V., Hungria V., Prausova J., Scagliotti G.V., Sleeboom H., Spencer A., Vadhan-Raj S. (2011). Randomized, double-blind study of denosumab versus zoledronic acid in the treatment of bone metastases in patients with advanced cancer (excluding breast and prostate cancer) or multiple myeloma. J. Clin. Oncol..

[81] Stopeck A.T., Lipton A., Body J.J., Steger G.G., Tonkin K., de Boer R.H., Lichinitser M., Fujiwara Y., Yardley D.A., Viniegra M. (2010). Denosumab compared with zoledronic acid for the treatment of bone metastases in patients with advanced breast cancer: A randomized, double-blind study. J. Clin. Oncol..

[82] Fizazi K., Carducci M., Smith M., Damião R., Brown J., Karsh L., Milecki P., Shore N., Rader M., Wang H. (2011). Denosumab versus zoledronic acid for treatment of bone metastases in men with castration-resistant prostate cancer: A randomised, double-blind study. Lancet.

[83] Henry D., Vadhan-Raj S., Hirsh V., von Moos R., Hungria V., Costa L., Woll P.J., Scagliotti G., Smith G., Feng A. (2014). Delaying skeletal-related events in a randomized phase 3 study of denosumab versus zoledronic acid in patients with advanced cancer: An analysis of data from patients with solid tumors. Supportive Care Cancer.

[84] Narayanan P. (2013). Denosumab: A comprehensive review. South Asian J. Cancer.

[85] Sisay M., Mengistu G., Edessa D. (2017). The RANK/RANKL/OPG system in tumorigenesis and metastasis of cancer stem cell: Potential targets for anticancer therapy. Onco Targets Ther..

[86] Dougall W.C. (2012). Molecular pathways: Osteoclast-dependent and osteoclast-independent roles of the RANKL/RANK/OPG pathway in tumorigenesis and metastasis. Clin. Cancer Res..

[87] Ming J., Cronin S.J.F., Penninger J.M. (2020). Targeting the RANKL/RANK/OPG Axis for Cancer Therapy. Front. Oncol..

[88] Kiechl S., Schramek D., Widschwendter M., Fourkala E.O., Zaikin A., Jones A., Jaeger B., Rack B., Janni W., Scholz C. (2017). Aberrant regulation of RANKL/OPG in women at high risk of developing breast cancer. Oncotarget.

[89] Chen L.M., Kuo C.H., Lai T.Y., Lin Y.M., Su C.C., Hsu H.H., Tsai F.J., Tsai C.H., Huang C.Y., Tang C.H. (2011). RANKL increases migration of human lung cancer cells through intercellular adhesion molecule-1 up-regulation. J. Cell. Biochem..

[90] Li X., Liu Y., Wu B., Dong Z., Wang Y., Lu J., Shi P., Bai W., Wang Z. (2014). Potential role of the OPG/RANK/RANKL axis in prostate cancer invasion and bone metastasis. Oncol. Rep..

[91] Van Poznak C., Somerfield M.R., Barlow W.E., Biermann J.S., Bosserman L.D., Clemons M.J., Dhesy-Thind S.K., Dillmon M.S., Eisen A., Frank E.S. (2017). Role of Bone-Modifying Agents in Metastatic Breast Cancer: An American Society of Clinical Oncology-Cancer Care Ontario Focused Guideline Update. J. Clin. Oncol..

[92] Saylor P.J., Rumble R.B., Tagawa S., Eastham J.A., Finelli A., Reddy P.S., Kungel T.M., Nissenberg M.G., Michalski J.M. (2020). Bone Health and Bone-Targeted Therapies for Prostate Cancer: ASCO Endorsement of a Cancer Care Ontario Guideline. J. Clin. Oncol..

[93] Campagnaro E., Reimers M.A., Qin A., Alva A.S., Schneider B.J., Van Poznak C.H. (2018). Use of Bone-Modifying Agents in Myeloma and Bone Metastases: How Recent Dosing Interval Studies Have Affected Our Practice. J. Oncol. Pract..

[94] Leng S., Lentzsch S. (2018). Bone-Modifying Agents: Complicated to Use. J. Oncol. Pract..

[95] Glantschnig H., Fisher J.E., Wesolowski G., Rodan G.A., Reszka A.A. (2003). M-CSF, TNFalpha and RANK ligand promote osteoclast survival by signaling through mTOR/S6 kinase. Cell Death Differ..

[96] Hadji P., Coleman R., Gnant M. (2013). Bone effects of mammalian target of rapamycin (mTOR) inhibition with everolimus. Crit. Rev. Oncol. Hematol..

[97] Lee R.J., Smith M.R. (2013). Targeting MET and vascular endothelial growth factor receptor signaling in castration-resistant prostate cancer. Cancer J..

[98] Liu C., Wang M., Xu C., Li B., Chen J., Chen J., Wang Z. (2021). Immune Checkpoint Inhibitor Therapy for Bone Metastases: Specific Microenvironment and Current Situation. J. Immunol. Res..

[99] Kimura T. (2018). Multidisciplinary Approach for Bone Metastasis: A Review. Cancers.

[100] Ibrahim T., Flamini E., Fabbri L., Serra P., Mercatali L., Ricci R., Sacanna E., Falasconi M.C., Casadei R., Galassi R. (2009). Multidisciplinary approach to the treatment of bone metastases: Osteo-Oncology Center, a new organizational model. Tumori J..

[101] Bongiovanni A., Recine F., Fausti V., Foca F., Casadei R., Falasconi M.C., Oboldi D., Sansoni E., Fabbri L., Micheletti S. (2019). Ten-year experience of the multidisciplinary Osteoncology Center. Supportive Care Cancer.

[102] Kuchuk I., Hutton B., Moretto P., Ng T., Addison C.L., Clemons M. (2013). Incidence, consequences and treatment of bone metastases in breast cancer patients-Experience from a single cancer centre. J. Bone Oncol..

[103] Lin P.P., Mirza A.N., Lewis V.O., Cannon C.P., Tu S.M., Tannir N.M., Yasko A.W. (2007). Patient survival after surgery for osseous metastases from renal cell carcinoma. J. Bone Jt. Surg..

[104] Ratasvuori M., Wedin R., Hansen B.H., Keller J., Trovik C., Zaikova O., Bergh P., Kalen A., Laitinen M. (2014). Prognostic role of en-bloc resection and late onset of bone metastasis in patients with bone-seeking carcinomas of the kidney, breast, lung, and prostate: SSG study on 672 operated skeletal metastases. J. Surg. Oncol..

[105] Tsukamoto S., Kido A., Tanaka Y., Facchini G., Peta G., Rossi G., Mavrogenis A.F. (2021). Current Overview of Treatment for Metastatic Bone Disease. Curr. Oncol..

[106] Lin S.C., Yu-Lee L.Y., Lin S.H. (2018). Osteoblastic Factors in Prostate Cancer Bone Metastasis. Curr. Osteoporos. Rep..

[107] Cho Y.J., Cho Y.M., Kim S.H., Shin K.-H., Jung S.-T., Kim H.S. (2019). Clinical analysis of patients with skeletal metastasis of lung cancer. BMC Cancer.

[108] D’Oronzo S., Coleman R., Brown J., Silvestris F. (2019). Metastatic bone disease: Pathogenesis and therapeutic options: Up-date on bone metastasis management. J. Bone Oncol..

[109] Ruatta F., Derosa L., Escudier B., Colomba E., Guida A., Baciarello G., Loriot Y., Fizazi K., Albiges L. (2019). Prognosis of renal cell carcinoma with bone metastases: Experience from a large cancer centre. Eur. J. Cancer.

[110] Umer M., Mohib Y., Atif M., Nazim M. (2018). Skeletal metastasis in renal cell carcinoma: A review. Ann. Med. Surg..

[111] Geraets S.E.W., Bos P.K., van der Stok J. (2020). Preoperative embolization in surgical treatment of long bone metastasis: A systematic literature review. EFORT Open Rev..

[112] Iñiguez-Ariza N.M., Bible K.C., Clarke B.L. (2020). Bone metastases in thyroid cancer. J. Bone Oncol..

[113] Strauss S.J., Frezza A.M., Abecassis N., Bajpai J., Bauer S., Biagini R., Bielack S., Blay J.Y., Bolle S., Bonvalot S. (2021). Bone sarcomas: ESMO-EURACAN-GENTURIS-ERN PaedCan Clinical Practice Guideline for diagnosis, treatment and follow-up. Ann. Oncol..

[114] Gatta G., Capocaccia R., Botta L., Mallone S., De Angelis R., Ardanaz E., Comber H., Dimitrova N., Leinonen M.K., Siesling S. (2017). Burden and centralised treatment in Europe of rare tumours: Results of RARECAREnet-a population-based study. Lancet Oncol..

[115] De Pinieux G., Karanian M., Le Loarer F., Le Guellec S., Chabaud S., Terrier P., Bouvier C., Batistella M., Neuville A., Robin Y.M. (2021). Nationwide incidence of sarcomas and connective tissue tumors of intermediate malignancy over four years using an expert pathology review network. PLoS ONE.

[116] World Health Organization (2020). Soft Tissue and Bone Tumours.

[117] Lalam R., Bloem J.L., Noebauer-Huhmann I.M., Wörtler K., Tagliafico A., Vanhoenacker F., Nikodinovska V.V., Sanal H.T., Woude H.V., Papakonstantinou O. (2017). ESSR Consensus Document for Detection, Characterization, and Referral Pathway for Tumors and Tumorlike Lesions of Bone. Semin. Musculoskelet. Radiol..

[118] Sweet D.E., Madewell J.E., Ragsdale B.D. (1981). Radiologic and pathologic analysis of solitary bone lesions. Part III: Matrix patterns. Radiol. Clin. N. Am..

[119] Ragsdale B.D., Madewell J.E., Sweet D.E. (1981). Radiologic and pathologic analysis of solitary bone lesions. Part II: Periosteal reactions. Radiol. Clin. N. Am..

[120] Monfardini L., Preda L., Aurilio G., Rizzo S., Bagnardi V., Renne G., Maccagnoni S., Vigna P.D., Davide D., Bellomi M. (2014). CT-guided bone biopsy in cancer patients with suspected bone metastases: Retrospective review of 308 procedures. Radiol. Med..

[121] Zustovich F., Pastorelli D. (2016). Therapeutic management of bone metastasis in prostate cancer: An update. Expert Rev. Anticancer Ther..

[122] Zhiyu W., Rui Z., Shuai W., Hui Z. (2016). Surgical treatment of patients with lung cancer and bone metastases: A prospective, observational study. Lancet.

[123] Roelofs A.J., Thompson K., Gordon S., Rogers M.J. (2006). Molecular mechanisms of action of bisphosphonates: Current status. Clin. Cancer Res..

[124] Luckman S.P., Hughes D.E., Coxon F.P., Graham R., Russell G., Rogers M.J. (1998). Nitrogen-containing bisphosphonates inhibit the mevalonate pathway and prevent post-translational prenylation of GTP-binding proteins, including Ras. J. Bone Miner. Res..

[125] Van der Linden Y.M., Kroon H.M., Dijkstra S.P., Lok J.J., Noordijk E.M., Leer J.W., Marijnen C.A. (2003). Simple radiographic parameter predicts fracturing in metastatic femoral bone lesions: Results from a randomised trial. Radiother. Oncol..

[126] Wernle J.D., Damron T.A., Allen M.J., Mann K.A. (2010). Local irradiation alters bone morphology and increases bone fragility in a mouse model. J. Biomech..

[127] Kitagawa Y., Ito T., Mizuno Y., Sudo Y., Kim Y., Tsunoda R., Takai S. (2019). Effect of Orthopedics Promotional Activities on Multidisciplinary Management of Patients with Bone Metastases. J. Nippon Med. Sch..

